# MicroRNA-Restricted Transgene Expression in the Retina

**DOI:** 10.1371/journal.pone.0022166

**Published:** 2011-07-26

**Authors:** Marianthi Karali, Anna Manfredi, Agostina Puppo, Elena Marrocco, Annagiusi Gargiulo, Mariacarmela Allocca, Michele Della Corte, Settimio Rossi, Massimo Giunti, Maria Laura Bacci, Francesca Simonelli, Enrico Maria Surace, Sandro Banfi, Alberto Auricchio

**Affiliations:** 1 Telethon Institute of Genetics and Medicine (TIGEM), Naples, Italy; 2 Medical Genetics, Department of Pediatrics, University of Naples Federico II, Naples, Italy; 3 Medical Genetics, Department of General Pathology, Second University of Naples, Naples, Italy; 4 Department of Veterinary Medical Science (DSMVET), University of Bologna, Bologna, Italy; 5 Department of Ophthalmology, Second University of Naples, Naples, Italy; University of Helsinki, Finland

## Abstract

**Background:**

Gene transfer using adeno-associated viral (AAV) vectors has been successfully applied in the retina for the treatment of inherited retinal dystrophies. Recently, microRNAs have been exploited to fine-tune transgene expression improving therapeutic outcomes. Here we evaluated the ability of retinal-expressed microRNAs to restrict AAV-mediated transgene expression to specific retinal cell types that represent the main targets of common inherited blinding conditions.

**Methodology/Principal Findings:**

To this end, we generated AAV2/5 vectors expressing *EGFP* and containing four tandem copies of miR-124 or miR-204 complementary sequences in the 3′UTR of the transgene expression cassette. These vectors were administered subretinally to adult C57BL/6 mice and Large White pigs. Our results demonstrate that miR-124 and miR-204 target sequences can efficiently restrict AAV2/5-mediated transgene expression to retinal pigment epithelium and photoreceptors, respectively, in mice and pigs. Interestingly, transgene restriction was observed at low vector doses relevant to therapy.

**Conclusions:**

We conclude that microRNA-mediated regulation of transgene expression can be applied in the retina to either restrict to a specific cell type the robust expression obtained using ubiquitous promoters or to provide an additional layer of gene expression regulation when using cell-specific promoters.

## Introduction

MicroRNAs (miRNAs) are a class of small 20–25-nucleotide long non-coding RNAs that negatively regulate expression of their target genes by binding to specific sequence elements in the 3′ untranslated region (UTR) of their respective mRNAs [1]. MiRNAs predominantly act to decrease target mRNA levels in animal cells [1] and at least one third of them are expressed in a cell type- or developmental-specific manner [2]. Only recently, endogenous miRNAs have been exploited for the tight post-transcriptional regulation of exogenously delivered (trans)genes in therapeutic and experimental applications [3]. Incorporation of target sites for a specific miRNA (miRTs) at the 3′ end of a transgene cassette has been adapted to provide a means of restricting transgene expression domains to specific cell types, lineages or differentiation states [4], [5], [6], [7], [8]. This strategy is particularly useful to further improve transgene specificity, when combined with the transcriptional targeting provided by tissue-specific promoters. A detailed knowledge of the cellular and developmental distribution of miRNAs is a major requisite towards the implementation of this strategy. In one of the first therapeutic applications of this approach, Brown and colleagues [9] combined an hepatocyte-specific promoter with target sequences for a hematopoietic-specific miRNA in a lentiviral-based vector to abolish the immune response generated by the off-target expression of clotting factor IX in the antigen-presenting cells (APCs) of Hemophilia B (ΔF.IX) mice. The dynamic expression of miRNAs has also been exploited to ensure an appropriate restriction of transgene expression across development [10].

Inherited retinal degenerations (IRDs) are a group of conditions that result from mutations in genes encoding proteins with critical functions in retinal pigment epithelium (RPE) or photoreceptor (PR) cells and lead to severe visual deficits and ultimately to blindness [11]. IRDs can greatly benefit from gene therapy using adeno-associated virus (AAV)-derived vectors that transduce non-dividing cells and result in long-term transgene expression [12]. The safety and efficacy of AAV-based gene therapy has been verified in various animal models [13] and in humans [14], [15], [16], [17], [18], [19], [20], [21]. Recently, AAV2-mediated gene transfer of *RPE65* in patients affected with Leber's Congenital Amaurosis (LCA, type 2; OMIM 204100) achieved stable improvement of visual and retinal function [14], [15], [16], [17], [18], [19], [20], [21].

Tight spatial and temporal control of transgene expression is desirable in the context of gene therapy. In IRDs, gene transfer should be ideally targeted to either RPE or PRs. This can be in part achieved by selecting the appropriate AAV serotype, as AAVs show variable kinetics of transgene expression and differential tropism for a broad range of ocular cell types [22]. Specificity of transgene expression in the retina can be further enhanced using RPE- or photoreceptor-specific promoter elements [23], [24]. However, very often the tissue-specific promoters used in gene therapy vectors do not faithfully recapitulate the patterns of the endogenous promoter. In addition, the levels of transgene expression obtained may either be inadequate for therapeutic purposes or supra-physiological and deleterious for retinal function.

With this study, we aimed to improve controlled transgene expression in the retina by exploiting the post-transcriptional regulation offered by the endogenous miRNA machinery. To this end, we integrated our knowledge on the cellular distribution of miRNAs within the mouse eye [25], [26] with the use AAV-mediated strategies for gene transfer to the retina [22]. Here we describe the first paradigm of harnessing retinal-specific miRNAs to delimit transgene expression to the RPE monolayer or the PRs of the adult retina using AAV vectors. We show that efficient restriction of transgene expression can be obtained even at low vector dosages. These findings have implications for the design of gene therapy approaches for hereditary retinopathies as they may improve safety and efficacy of gene transfer.

## Results

### Use of miR-204 target sites restricts transgene expression to murine PRs *in vivo*

**Figure 1:**
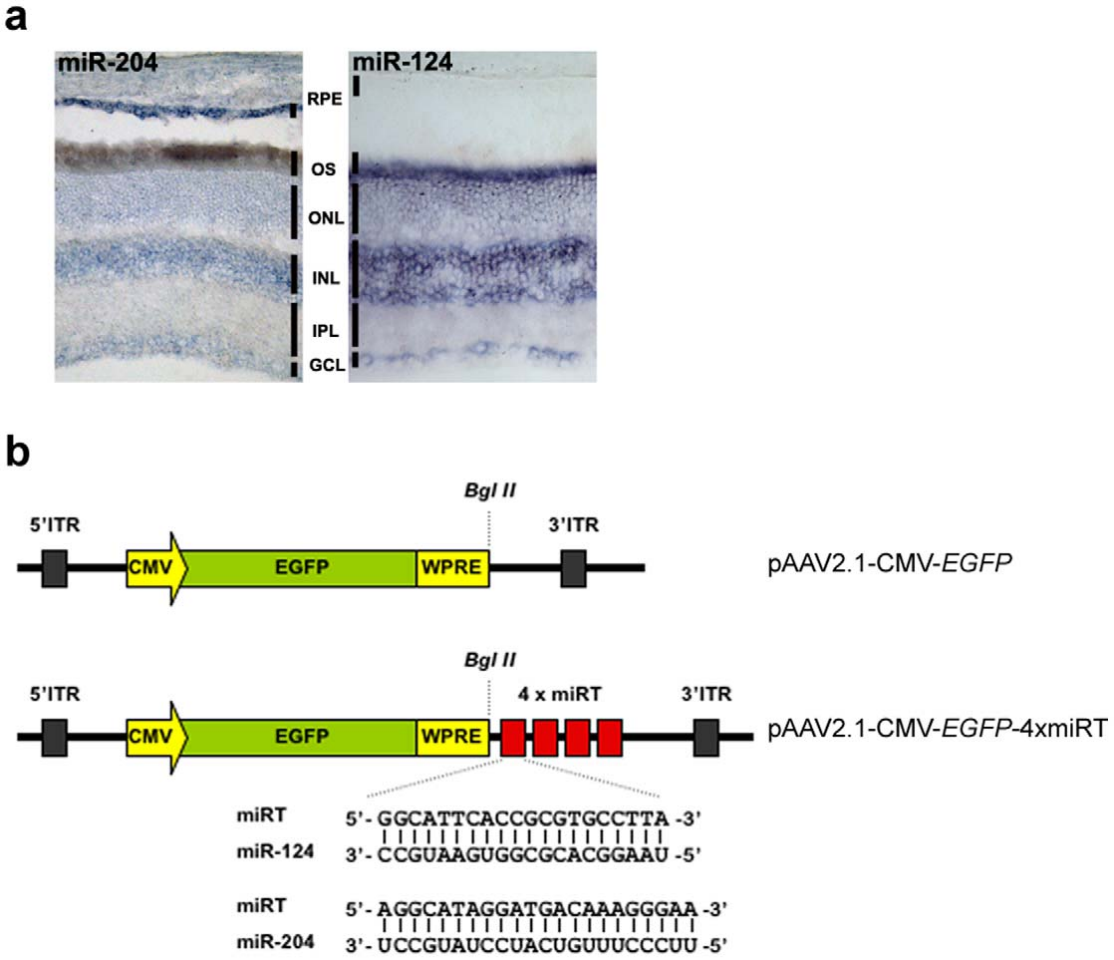
miR-124 and miR-204 expression in retina supports the use of AAV vectors harboring corresponding miRTs. (**a**) Expression profile of miR-204 and miR-124 in retina sections of adult, albino (CD1) mouse as revealed by ISH using LNA-modified probes. miR-124, a neuronal-specific miRNA, is expressed in all layers of the neural retina but is not detected in the RPE. miR-204 is expressed in the GCL and the INL of the neural retina and stains strongly the RPE. RNA ISH for the detection of mature miRNAs was performed using miRCURY LNA™ microRNA Detection Probes (Exiqon, Vedbaek, Denmark) as previously described [26]. (**b**) Schematic representation of the AAV vectors harboring the miRT sites. Four tandem copies (4xmiRT) of a sequence perfectly complementary to the sequence of the mature miR-124 or miR-204 (see alignments) were cloned downstream of the WPRE element in the pAAV2.1-CMV-*EGFP* plasmid. Abbreviations are as follows: CMV, Human Cytomegalovirus promoter; EGFP, Enhanced Green Fluorescence Protein; GCL, Ganglion Cell Layer; INL, Inner Nuclear Layer; IPL, Inner Plexiform Layer; ITR, Inverted Terminal Repeat; miRT, miRNA target site; ONL, Photoreceptor Outer Nuclear Layer; OS, Photoreceptor Outer Segments; RPE, Retinal Pigment Epithelium; WPRE, Woodchuck hepatitis virus Post-transcriptional Regulatory Element.

We sought to assess whether post-transcriptional restriction of AAV-mediated transgene expression to PRs could be achieved by exploiting endogenous miRNAs. For this purpose we selected miR-204, a miRNA that is strongly expressed in the RPE from as early as E10.5 to adulthood [25], [26] (**Figure 1a**). miR-204 expression was absent from the PRs and was detected by *in situ* hybridization (ISH) at low levels in the inner nuclear layer of the neural retina as well in the ganglion cell layer [26] (**Figure 1a**).

Based on the miR-204 expression pattern in the adult murine retina, we inserted four copies of a sequence that is perfectly complementary to the mature miR-204 (miR204T) immediately downstream of the Woodchuck hepatitis virus Post-transcriptional Regulatory Element (WPRE) in the pAAV2.1-CMV-*EGFP* plasmid (**Figure 1b**; see Materials and Methods). The presence in this plasmid of the ubiquitous CMV promoter drives robust transgene expression in both RPE and PRs [27], [28]. The resulting pAAV2.1-CMV-*EGFP*-4xmiR204T plasmid (**Figure 1b**) was used to produce AAV2/5 vectors that efficiently transduce RPE and PRs upon subretinal administration in several species, including mice [29]. Four-week-old C57BL/6 mice (n = 4) were injected with 2.6×10^9^ genome copies (GC) (defined as “high dose”) of AAV2/5-CMV-*EGFP*-4xmiR204T in one eye, and the same dose of AAV2/5-CMV-*EGFP* as control in the contralateral one. Four weeks after injection, eyes were harvested, retinas were sectioned and retinal sections were analyzed by direct fluorescence microscopy to assess localization of EGFP expression.

**Figure 2:**
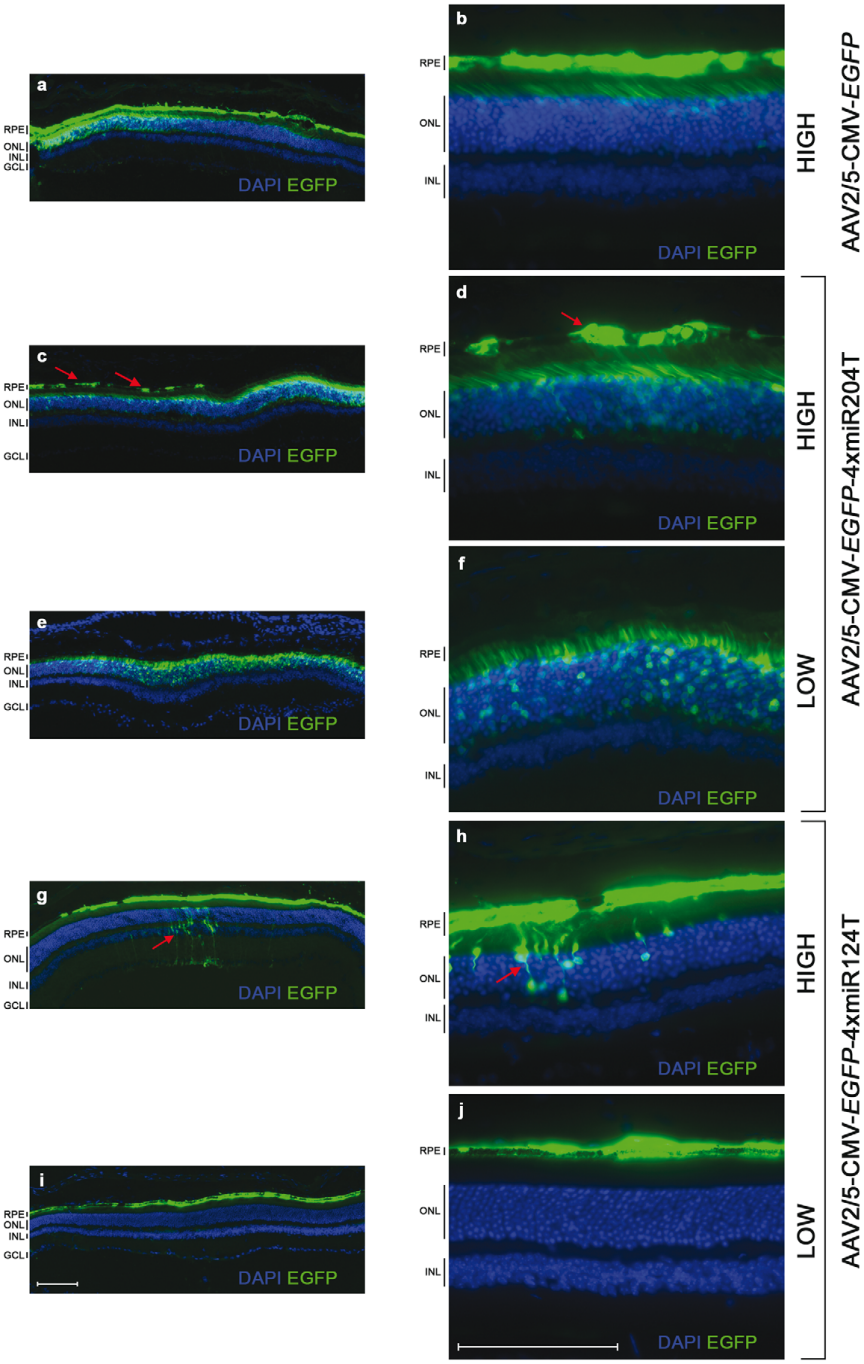
miRNA-regulated *EGFP* expression in the mouse retina. C57BL/6 adult mice (n = 4 eyes/group) were injected subretinally with: 2.6×10^9^ GC/eye of AAV2/5-CMV-*EGFP* (**a** and **b**; high dose); 2.6×10^9^ GC/eye (**c** and **d**; high dose) or 2.6×10^8^ GC/eye (**e** and **f**; low dose) of AAV2/5-CMV-*EGFP*-4xmiR204T; 2.6×10^9^ GC/eye (**g** and **h**; high dose) or 2.6×10^8^ GC/eye (**i** and **j**; low dose) of AAV2/5-CMV-*EGFP*-4xmiR124T. Mice were sacrificed four weeks after injection, and retinal sections were analyzed by direct fluorescence microscopy. Images at 10X (panels on the left) and 40X magnification (panels on the right) are shown. At high vector doses ectopic EGFP expression (red arrows) was observed in few PR and RPE cells, respectively. Scale bar = 100 µm. Abbreviations: RPE, retinal pigment epithelium; ONL, photoreceptor outer nuclear layer; INL, inner nuclear layer; GCL, ganglion cell layer.

The number of EGFP-positive RPE cells in the eyes injected with AAV2/5-CMV-*EGFP*-4xmiR204T (**Figures 2c,d**) was dramatically reduced, compared to contralateral eyes injected with AAV2/5-CMV-*EGFP* (**Figures 2a,b**), indicating efficient suppression of the *EGFP*-miR204T mRNA by endogenous miR-204. Despite the strong reduction of EGFP expression in the RPE, occasional EGFP-positive RPE cells were detected (red arrows in **Figures 2c,d**). We hypothesized that loss of miR-204-mediated regulation in these EGFP-positive RPE cells could result from the saturation of the miRNA activity due to an excess of exogenous miR204Ts. To indirectly test this, we used a 10-fold lower vector dose (2.6×10^8^ GC/eye; defined as “low dose”). Analysis of EGFP fluorescence in the low-dose group (n = 4) showed specific restriction of transgene expression to the PRs, while no EGFP expression could be detected within the RPE in any of the sections from the four eyes injected with the miR204T-containing vector (**Figures 2e,f**).

### Use of miR-124 target sites restricts transgene expression to murine RPE *in vivo*

To restrict AAV2/5-CMV-mediated transgene expression to the RPE, we exploited miR-124, a miRNA abundantly expressed in differentiated neurons [30]. We and others have shown by ISH that miR-124 stains strongly all retinal cell layers, but is not detected in the RPE [26], [31] (**Figure 1a**). Therefore, we cloned four tandem copies of a sequence that is perfectly complementary to the mature miR-124 downstream of the WPRE element in the pAAV2.1-CMV-*EGFP* plasmid (**Figure 1b**). The resulting pAAV2.1-CMV-*EGFP*-4xmiR124T plasmid was used to produce AAV2/5 vectors that were administered to C57BL/6 mice by subretinal injection. Mice (n = 4) received 2.6×10^9^ GC (defined as “high dose”) of AAV2/5-CMV-*EGFP*-4xmiR124T in one eye, and the same dose of the AAV2/5-CMV-*EGFP* as control in the contralateral one. The animals were sacrificed four weeks after injection. Reporter expression in the transduced retina was evaluated by fluorescence microscopy of retinal sections.

We observed a dramatic reduction in the number of EGFP-positive PRs in eyes injected with the AAV2/5-CMV-*EGFP*-4xmiR124T (**Figures 2g,h**) compared to eyes injected with the control vector (**Figures 2a,b**), suggesting efficient elimination of the miRT-containing transcript by the endogenous miR-124. However, similarly to what observed with the miR204T-containing construct, few scattered EGFP-positive PR cells could be seen in the neural retina (red arrows in **Figures 2g,h**), which implies loss of miRNA-mediated regulation therein. EGFP expression in these cells could result from the saturation of miRNA activity due to an excess of exogenously provided miR124Ts. We then tested a 10-fold decrement in vector dose to assess if off-target expression of the miRT-containing transcript in the transduced PRs would be eliminated. C57BL/6 mice (n = 4) were injected with 2.6×10^8^ GC/eye of either virus, and their eyes were analyzed four weeks after injection. We did not observe any EGFP-positive cells in the neural retina of eyes administered with the low dose of AAV2/5-CMV-*EGFP*-4xmiR124T (**Figures 2i,j**), suggesting that at this dose, the presence of the miR124Ts tightly restricts transgene expression to the RPE.

Finally, to exclude that the presence of exogenous miRNA target sequences can interfere with the physiological function of the PRs, we performed electroretinograms (ERG) on mice injected at a high dose (2.6×10^9^ GC/eye) with the AAV2/5 vectors harboring the miR-124 or miR-204 target sequences and the control *EGFP* construct. ERG recordings of eyes injected with the miRT-bearing vectors showed no statistically significant differences compared to eyes injected with the *EGFP* control [max a-wave amplitude: EGFP = 336,18 µV (±67,06); miR124T = 350,97 (±132,11) µV; miR204T = 320,70 (±105,09) µV; max b-wave amplitude: EGFP = 667,83 (±165,47) µV; miR124T = 598,18 (±90,28) µV; miR204T = 690,83 (±143,96) µV].

### miRNA-regulation of transgene expression in the porcine retina

We extended our studies to the pig (*Sus scrofa*) as, among non-primate mammals, the porcine retina most closely resembles the human one in terms of size, anatomy, cellular composition and physiology, rendering it a valuable preclinical model system for eye disease and therapy [32]. The mature sequence of miR-124 and miR-204 is identical in pigs and mouse (miRBase, http://www.mirbase.org/; [33]). Given the highly conserved cellular distribution of these two miRNAs across species [26], [34], [35], we assumed that miR-124 and miR-204 are likely to be expressed in the same porcine retinal layers.

**Figure 3:**
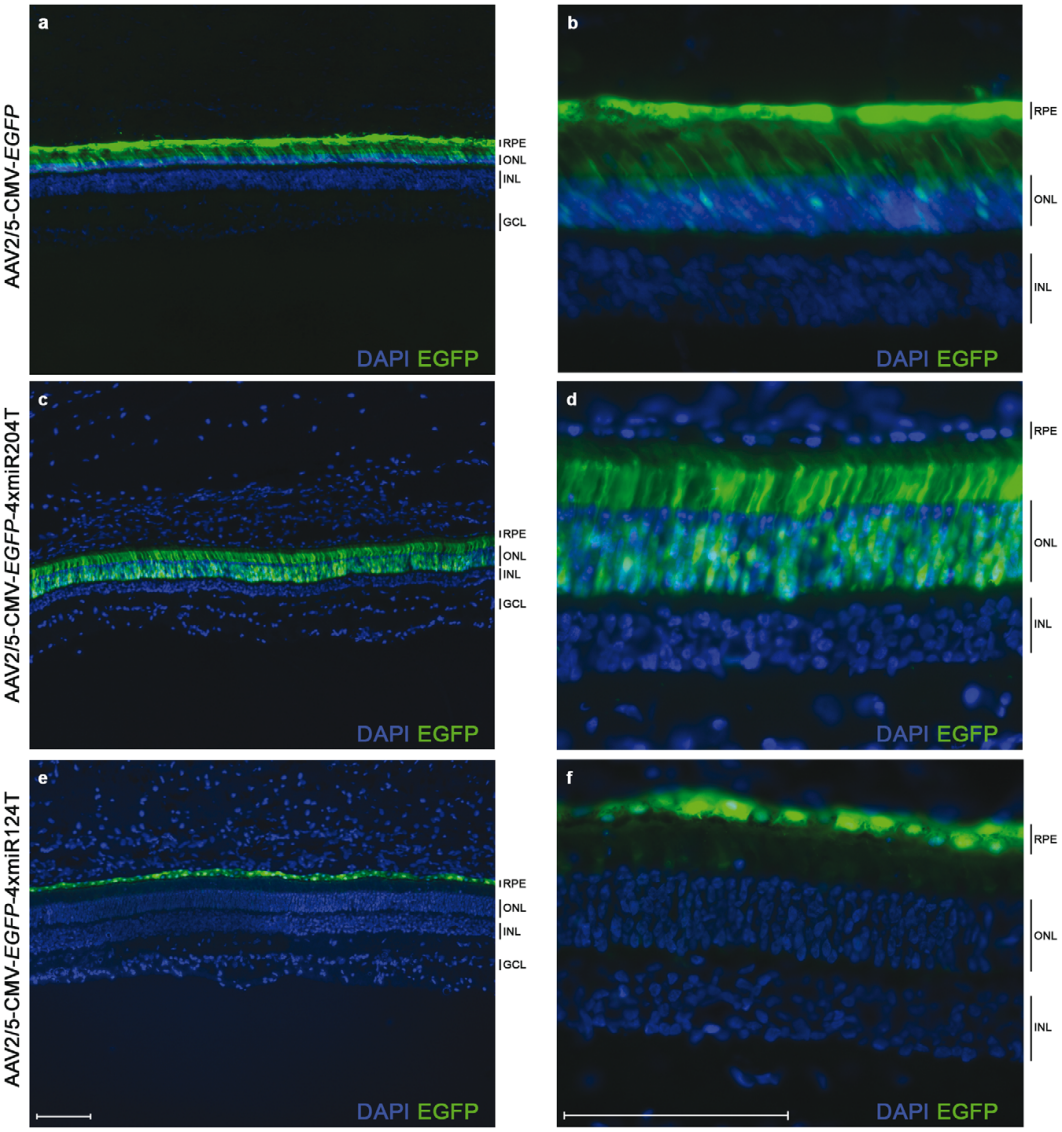
miRNA-regulated *EGFP* expression in the pig retina. Large White (LW) female pigs (n = 2 eyes/group) were injected subretinally with: AAV2/5-CMV-*EGFP* (**a** and **b**); AAV2/5-CMV-*EGFP*-4xmiR204T (**c** and **d**); AAV2/5-CMV-*EGFP*-4xmiR124T (**e** and **f**). All eyes were injected with 1×10^10^ GC/eye of each vector. Retinal cryosections were obtained six weeks after injection and analyzed by direct fluorescence microscopy. Magnification 10X (**a–c**) and 40X (**d–f**). Scale bar = 100 µm. For abbreviations, see Fig. 2 legend.

We injected subretinally eleven week-old Large White (LW) female pigs (n = 2 eyes/group) with AAV2/5-CMV-*EGFP*-4xmiR204T and AAV2/5-CMV-*EGFP*-4xmiR124T and compared them with eyes injected with the AAV2/5-CMV-*EGFP* as control. Considering the size proportions of murine and porcine eyes, the dose administered in pigs was equivalent to the low dose injected in mouse (in the mouse eye we administered 1 µl containing 2.6×10^8^ GC, in the pig eye 100 µl of a 1∶2.6 dilution of the same vector solution, thus containing 1×10^10^ GC). Retinal sections were analyzed, following animal sacrifice six weeks after injection. As shown in Figure 3, the use of target sequences for miR-204 (**Figures 3c,d**) and miR-124 (**Figures 3e,f**) efficiently restricted AAV2/5 mediated *EGFP* expression to the PRs and RPE of the porcine retina, respectively.

**Figure 4:**
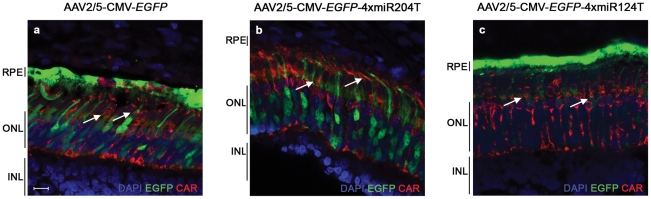
Cone transduction following subretinal administration of AAV vectors bearing miRNA target sites. Retinal cryosections from animals injected subretinally with the various miRNA-regulated vectors as in Fig. 3 were immunolabelled with the cone-specific anti-Cone Arrestin (CAR) antibody (red label). Representative sections of eyes injected with AV2/5-CMV-*EGFP* (**a**), AAV2/5-CMV-*EGFP*-4xmiR204T (**b**) and AAV2/5-CMV-*EGFP*-4xmiR124T (**c**). Colocalization of EGFP and CAR expression is indicated by the arrows. Confocal microscope magnification 63X. Scale bar = 10 µm. For abbreviations, see Fig. 2 legend.

Cones are important targets of gene therapy since several blinding conditions, either inherited as monogenic or as complex traits, are due to mutations in genes expressed in cones or are characterized by progressive cone degeneration [36]. Since the porcine retina has a high number of cones compared to the murine one [37], we checked whether both rod and cone PRs were equally transduced following AAV2/5-mediated delivery. Expression of EGFP in cone PRs was confirmed by immunolabelling of porcine retinal sections with the Cone Arrestin antibody (anti-hCAR) (arrows in **Figure 4**). A weak EGFP fluorescence was detected by confocal microscopy in the PRs of eyes injected with the AAV2/5-CMV-*EGFP*-4xmiR124T (**Figure 4c**).

**Figure 5:**
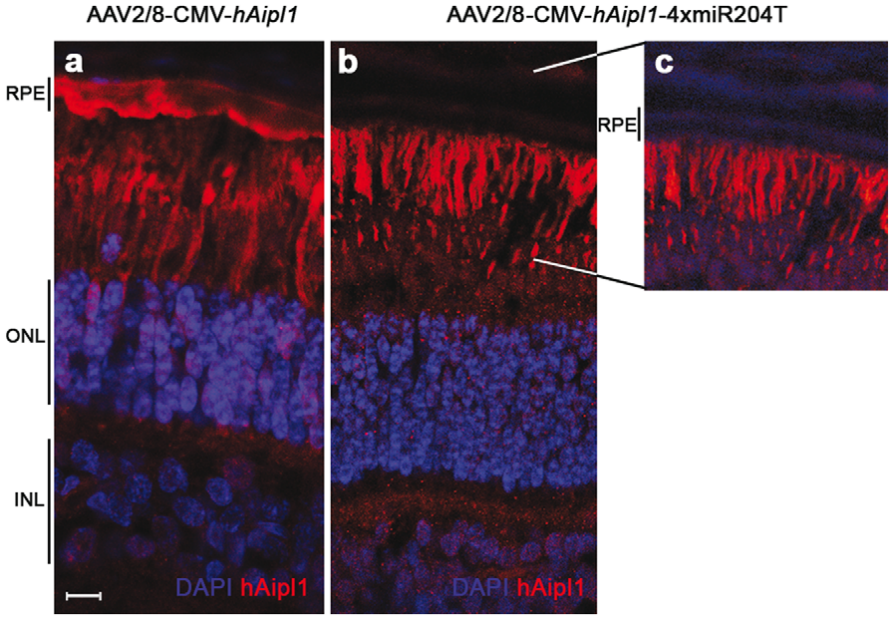
miR204-regulated expression of *hAIPL1* is restricted to the porcine photoreceptors. LW pigs were injected with 1×10^10^ GC/eye with AAV2/8-CMV-*hAIPL1* (**a**) and AAV2/8-CMV-*hAIPL1*-4xmiR204T (**b**) (n = 2 eyes/group). Human AIPL1 immunostaining (red) was performed on pig retinal sections to assess the localization of the transgene expression. The antibody used does not cross-react with the porcine Aipl1. An over-exposure of the section in panel (**b**) is shown in (**c**) to highlight that no *hAIPL1* expression was detected in the RPE cells of eyes injected with AAV2/8-CMV-*hAIPL1*-4xmiR204T. Confocal microscope magnification 63X. Scale bar = 10 µm. For abbreviations, see Fig. 2 legend.

With the prospect of using this strategy in animal models of inherited retinal degeneration, we included target sequences for miR-204 in an AAV vector encoding the human *AIPL1* gene, mutated in LCA type 4 (OMIM 604393), with the aim to efficiently transfer *AIPL1* to PRs, its main expression site in the retina. We have recently shown that AAV2/8 vectors target murine [27] and porcine PRs [38] more efficiently than AAV2/5. Therefore, we generated an AAV2/8-CMV-*hAIPL1*-4xmiR204T vector. The AAV2/8-CMV-*hAIPL1*-4xmiR204T vector was injected subretinally in two eleven week-old Large White (LW) female pigs along with the control AAV2/8-CMV-*hAIPL1* that lacks the miR-204 target sites in the contralateral eye. The animals were sacrificed six weeks after injection and their eyes were harvested. Retinal cryosections were then analyzed by immunofluorescence with antibodies directed to human, but not porcine, AIPL1 using confocal microscopy. Transgene expression was detected in both PRs and RPE of the porcine retinas injected with the AAV2/8-CMV-*hAIPL1* control vector, while hAIPL1 expression was efficiently restricted to the PRs in retinas injected with the miR204-regulated vector (**Figure 5**).

To assess whether the presence of miRNA target sequences perturbs normal retinal function, ERG recordings were performed on all injected pigs. Both rod and cone isolated and combined responses of treated eyes (n = 2/vector) showed no statistically significant differences compared to baseline measurements [baseline pre-treatment, photopic = 120 (±4,86) µV; scotopic = 44,7 (±0,5) µV and maximal response 196,6 (±9,21) µV; post-treatment, photopic = 130,5 (±5,5) µV; scotopic = 48 (±2,5) µV and maximal 202 (±5) µV] indicating normal retinal function in the injected animals.

### Subretinal administrations of AAV vectors harboring miRNA target sites do not significantly perturb endogenous miRNA activity in the eye

As described before, we detected some scattered and discontinuous EGFP-positive areas within the RPE and PR layers (red arrows in **Figures 2c,d,g,h**) of murine retinas injected with high doses (2.6×10^9^ GC/eye) of AAV2/5-CMV-*EGFP*-4xmiR204T and AAV2/5-CMV-*EGFP*-4xmiR124T, respectively. We hypothesized that endogenous miRNAs did not completely eliminate the miRT-bearing EGFP transcripts, thus resulting in unexpected EGFP expression. This could be due to miRNA saturation (*i.e*. depletion of levels of the corresponding endogenous miRNA) caused by the presence of an excessive number of exogenously provided miRNA binding sites. Alternatively, the high number of vector-borne miRTs may have elicited a general deregulation of the miRNA/RISC machinery, resulting in a compromised repressive function. When mice were injected with the same vectors at lower doses, we did not detect any ectopic EGFP fluorescence (**Figures 2e,f,i,j**), suggesting that off-target transgene expression was a dosage-dependent phenomenon.

To assess whether expression of exogenous sequences carrying miRTs could interfere with the normal function of the miRNA machinery, we analyzed the endogenous levels of the corresponding miRNAs by qRT-PCR. In particular, we analyzed the expression levels of miR-204 and miR-124, as well as of miR-182. The latter is strongly expressed in the neural retina, predominantly in PRs [25], [26], and, to our current knowledge, is unlikely to either be under the direct control of any of the miRNAs tested or have affinity for the miR-204 and miR-124 miRTs. We extracted total RNA either from retinas of AAV2/5-CMV-*EGFP*-4xmiR124T injected mice (n = 4; high dose group) or from the optic cups (including retina, RPE and sclera) of animals injected with AAV2/5-CMV-*EGFP*-4xmiR204T (n = 4; high dose group). As miR-204 is mainly expressed in the RPE and off-target EGFP expression was detected therein, we reasoned that any potential saturation of miR-204 should be assessed in samples that include RPE. We did not observe any significant variation in the endogenous levels of the miRNAs analyzed (**Figure S1a**), implying the absence of a detectable miRNA saturation effect at the level of the whole retinas or optic cups.

To check whether the high number of miRTs could perturb the capacity of miRNAs to regulate their physiological target genes, we analyzed the expression levels of *VAMP3* and *RDH10*, two direct targets of miR-124 [39], [40] in retinal samples of animals (n = 4) injected with high doses of either the control AAV2/5-CMV-*EGFP* or the construct bearing the miR-124 binding sites. VAMP3, also known as cellubrevin, is a member of the vesicle-associated membrane protein (VAMP)/synaptobrevin family [41] and is strongly expressed in the retina, according to the BioGPS gene annotation portal (http://biogps.gnf.org/). On the other hand, *RDH10* encodes for retinol dehydrogenase 10 (all-trans) and is primarily expressed in the RPE and the neural retina [42](Mouse Retina SAGE library; [43]). We did not detect any significant variation in the endogenous levels of these target genes (**Figure S1b**), suggesting that the AAV-borne miR124Ts do not interfere with normal miRNA function. Taken together, the above results imply that the exogenously supplied miRTs do not saturate endogenous miRNA levels nor alleviate miRNA repression from its natural targets.

## Discussion

Recently, miRNA-mediated regulation of transgene expression has been successfully achieved in the context of somatic gene transfer in specific cell types, lineages or developmental stages [3]. In the present study, we show for the first time that this strategy can be applied to the retina. Subretinal administration of AAV2/5 results in optimal RPE transduction, but also in robust transgene expression in PRs [22]. Here, we demonstrate that subretinal administration of AAV2/5 vectors containing expression cassettes harboring binding sites for miRNAs expressed in specific retinal cell types results in transgene expression tightly restricted to either the RPE or PRs of mice and pigs. In particular, constructs harboring miR-124 binding sites can efficiently de-target reporter expression from the PRs, while the presence of miR-204 sites induces elimination of transgene expression from the RPE. The validation of this approach in the porcine retina confirmed its high clinical potential.

The use of miRNA-regulated vectors can be advantageous in gene therapy for inherited eye diseases: in one instance, when coupled to cell-specific promoters, it adds a layer of regulation to transgene expression. This is desirable as often the promoter elements used in gene therapy vectors do not faithfully recapitulate the expression patterns of the endogenous promoter, probably due to the absence of distant, secondary regulatory elements [9]. For instance, the promoter of *Rhodopsin* - a gene strongly and specifically expressed in rod PRs- induces aberrant reporter expression also in cones [27], [44]. In addition, the levels of transgene expression obtained using tissue-specific promoters may not be adequate. For instance, the *Rhodopsin* promoter drives very robust gene expression in PRs. This is not surprising since rhodopsin accounts for more than 70% of PR proteins [45]. While the Rhodopsin promoter element may be desirable if one wants to replace or repress *Rhodopsin* expression, the levels of expression of other transgenes may be supra-physiological or toxic. On the other hand, other retinal-specific promoters, like *RPE65* [23] or *OA1* [46], may provide subtherapeutic levels of transgene expression for some applications in the RPE. Indeed, in one LCA2 clinical trial (ClinicalTrials.gov number, NCT00516477), we have used AAV2/2 vectors expressing *RPE65* from the robust constitutive CBA promoter to obtain therapeutic levels of trangene expression. Assuming that the CBA expression pattern following subretinal administration of AAV2/2 vectors in the human retina is similar to that observed in mice and dogs [47], [48], we are presumably misexpressing the *RPE65* in PRs in addition to the RPE. Although this does not appear to be a problem as we did not observe any retinal toxic effect so far [21], ideally we could have tailored *RPE65* expression to RPE if we had included miR-124 target sites in our AAV vector.

A main concern for the application of miRNA-regulated transgene delivery is the potential to saturate their cognate miRNA by exogenously provided miRTs, thus reducing its bioavailability and alleviating control of its natural targets. Interestingly, in all studies that have employed lentiviral platforms for the delivery of miRNA-regulated transgenes, there is no evidence that the excess of exogenously provided miRTs - when engineered according to the principles set for regulated targets [3] - saturates the cognate miRNA [3], [4], [5], [6], [9]. Gentner and colleagues showed that only when expression is driven by very strong promoters or when several vector copies are introduced, transgene constructs containing four copies of perfectly complementary miRTs are able to saturate miRNA regulation following lentiviral delivery [49]. In this study, we designed our vectors according to the principles set for regulated targets to prevent miRNA inhibition in the presence of a strong CMV promoter [3]. Indeed, most of the studies applying these parameters report no evidence of miRNA saturation [3]. We observed some scattered, off-target EGFP expression in murine retinas injected with high doses of miRT-bearing vectors, indicative of miRNA inhibition which was not detected when using a ten-fold lower AAV vector dose. However, we did not measure altered levels of neither miRNAs nor their target genes in the retinas treated with high AAV vector doses suggesting that miRNA saturation, if present, occurs at levels below the detection limit of our assay. As an alternative to lowering the dose of viral genome copies administered, the number of miRTs present in the vector construct could be reduced.

The PR- and RPE-restricted pattern of transgene expression at low vector doses was confirmed in the pig retina, which is more similar to the human one in terms of size and anatomy [32]. We can thus expect that a dose similar to the one successfully used in pigs could be applied to humans. Indeed, we have used a similar dose (1.5×10^10^ GC/eye) of AAV2/2 in patients with LCA2 obtaining improvement of visual function [19], [20], [21]. Our data suggest that administration of low doses of miRT-harboring vectors enables to tailor transgene expression to specific retinal cell types in the absence of off-target effects and deregulation of endogenous miRNA activity.

Ultimately, the efficacy of AAV-mediated miRNA-regulated gene expression in the retina should be proven in animal models of IRDs and, ideally, in non-human primates which possess a cone-enriched macula. Our data suggest that the addition of miRNA target sites to gene therapy vectors enables fine-tuning of transgene expression in the retina possibly rendering gene therapy safer and more efficient.

## Materials and Methods

### Plasmid construction, AAV vector production and purification

Recombinant AAV vectors containing the EGFP cDNA under the cytomegalovirus (CMV) promoter and four copies of a sequence (referred to as ‘miRT’) that is perfectly complementary to the miRNAs of interest in the 3′ UTR were constructed by a two-step cloning protocol. Initially, the cassette containing four copies of a sequence which is perfectly complementary to miR-204 (in capital letters) was constructed by annealing the following two sets of oligonucleotides: 5′-ctagatctAGGCATAGGATGACAAAGGGAAcgataggcatAGGATGACAAAGGGAAaagctt-3′, 5′-TTCCCTTTGTCATCCTATGCCTatcgTTCCCTTTGTCATCCTATGCCTAGAT-3′ and 5′-AGGCATAGGATGACAAAGGGAAtcacAGGCATAGGATGACAAAGGGAAagatc-3′, 5′-tcgagatctTCCCTTTGTCATCCTATGCCTgtgaTTCCCTTTGTCATCCTATGCCTaagctt-3′.

Similarly, the cassette containing four copies of a sequence which is perfectly complementary to miR-124 (in capital letters) was constructed by annealing the following two sets of oligonucleotides:

5′-ctagatctGGCATTCACCGCGTGCCTTAcgatGGCATTCACCGCGTGCCTTAaagctt-3′, 5′-TAAGGCACGCGGTGAATGCCatcgTAAGGCACGCGGTGAATGCCagat-3′ and ′-GGCATTCACCGCGTGCCTTAtcacGGCATTCACCGCGTGCCTTAagatc-3′5, 5′-tcgagatctTAAGGCACGCGGTGAATGCCgtgaTAAGGCACGCGGTGAATGCCaagctt-3′. In either case, the resulting double-stranded fragments (each one containing two copies of the respective miRT) were ligated thanks to the presence of phosphorylated 5′ ends. The obtained fragments (containing four copies of the respective miRT) were subcloned in pBluescript II SK(+) previously digested with *Xba* I and *Xho* I. The recombinant clones were digested with *Bgl* II to release the fragment containing the four miRT sites with *Bgl* II protruding ends. The fragment was then cloned into the *Bgl* II site of the pAAV2.1-CMV-*EGFP* plasmid [27] and used for the production of AAV2/5 vectors.

To generate the vectors expressing *hAIPL1*, the coding sequence of the *hAIPL1* gene was amplified from human retina cDNA (BioChain, Hayward, CA) using the primers hAIPL1-NotI-forward (5′-ATATGCGGCCGCCATGGATGCCGCTCTGCTCCT- 3′) and hAIPL1-HindIII-reverse (5′- ACGCGTAAGCTTTTATCAGTGCTGCAGCGAGTGCC- 3′) and cloned into the pAAV2.1-CMV-*EGFP* following digestion with *Not* I and *Hind* III. The final pAAV2.1-CMV-*hAIPL1*-4xmiR204T plasmid was subsequently produced by cloning the fragment containing four miR-204 target sites (released by *Bgl* II digestion of the pAAV2.1-CMV-*EGFP*-4xmiR204T) in the *Bgl* II site of pAAV2.1-CMV-*hAIPL1*.

AAV vectors were produced by triple transfection of 293 cells, purified by two rounds of CsCl_2_ ultracentrifugation, and titered (in GC/milliliter) using a real-time PCR-based assay TaqMan® (Applied Biosystems, Foster City, CA) and a dot-blot analysis, as previously described [28]. AAV vector production was carried out by the TIGEM AAV vector core.

### Animal procedures and vector administration

#### Ethics Statement

All studies on mice were conducted in strict accordance with the institutional guidelines for animal research and with the Association for Research in Vision and Ophthalmology (ARVO) Statement for the Use of Animal in Ophthalmic and Vision Research. All animal treatments were reviewed and approved in advance by the Ethics Committee of the Centre of Biotechnology, Animal Research Unit, Cardarelli Hospital (Naples, Italy). All procedures on mice were then approved by the Italian Ministry of Health (protocol number: 0000667/11/CB; approval date Sept. 11, 2007).

The experiments involving pigs were conducted according to relevant national and international guidelines. All procedures on pigs were reviewed and approved in advance by the Ethics Committee of the Department of Veterinary Medical Science, University of Bologna (Bologna, Italy) and were then approved by the Italian Ministry of Health (protocol number: 23/2009-B, approval date Feb. 04, 2009). All surgery was performed under anesthesia, and all efforts were made to minimize suffering.

#### Mice

Four-week old C57BL/6 mice (Harlan, S. Pietro al Natisone, Italy) were anesthetized with an intraperitoneal injection of avertin (1.25% w/v of 2,2,2-tribromoethanol and 2.5% v/v of 2-methyl-2-Butanol; Sigma–Aldrich, St. Louis, MO) at 2 ml/100 g of body weight, and viral vectors were delivered via a trans-scleral transchoroidal approach, as previously described [50]. Mice were injected in the right eye with 2.6×10^9^ GC of AAV2/5-CMV-*EGFP*-4xmiRT in the high dose experiments and 2.6×10^8^ GC of AAV2/5-CMV-*EGFP*-4xmiRT in the low dose. The same doses of AAV2/5-CMV-*EGFP* were injected in the left eye, as control. Following injection, the extent of transduction was assessed by ophthalmoscopy at days 7 and 28. Eyes were harvested at day 28 after injection.

#### Pigs

The Large White pigs (LW) used in our study were registered as purebred in the LW Herd Book of the Italian National Pig Breeders' Association. Pigs were starved overnight leaving water *ad libitum*. The anesthetic and surgical procedures for pigs were previously described [38].

### Histological analysis

Mice were sacrificed, and their eyeballs were then harvested and fixed overnight by immersion in 4% paraformaldehyde (PFA). Before harvesting the eyeballs, the temporal aspect of the sclerae was marked by cautery in order to orient the eyes with respect to the injection site at the moment of the inclusion. The eyeballs were cut so that the lens and vitreous could be removed leaving the eyecup intact. Mice eyecups were infiltrated with 30% sucrose for cryopreservation, and embedded in tissue freezing medium (O.C.T. matrix, Kaltek, Padua, Italy). For each eye, 150 to 200 serial sections (10 µm-thick) were cut along the horizontal plane and the sections were progressively distributed on 10 slides so that each slide contained 15 to 20 sections, each representative of the whole eye at different levels. The sections were stained with 4′,6′-diamidino-2-phenylindole (Vectashield, Vector Lab Inc., Peterborough, UK) and EGFP was monitored with a Zeiss Axiocam (Carl Zeiss, Oberkochen, Germany) at different magnifications.

Pigs were sacrificed and their eyeballs were harvested and fixed overnight by immersion in 4% PFA. The eyeballs were cut so that the lens and vitreous could be removed, leaving the eyecups in place. The eyecups were cryoprotected by progressive infiltration with 10%, 20% and 30% sucrose. Before embedding, the swine eyecups were analyzed under a fluorescence stereomicroscope (Leica Microsystems GmbH, Wetzlar, Germany) in order to localize the transduced region, whenever an EGFP-encoding vector was administered. Embedding was performed in tissue-freezing medium (O.C.T. matrix, Kaltek, Padua, Italy). For each eye, 200 to 300 serial sections (12 mm-thick) were cut along the horizontal meridian and the sections were progressively distributed on glass slides so that each slide contained 6 to 10 sections. Section staining and image acquisition was performed as described for mice.

### Immunofluorescence staining

Frozen retinal sections were washed once with PBS and then fixed for 10 min in 4% PFA. Sections were then permeabilized for 1 hour in PBS containing 0.1% Triton® X-100. Blocking solution containing 10% normal goat serum (Sigma–Aldrich, St. Louis, MO) was applied for 1 hour. Primary antibodies were diluted in PBS and incubated overnight at 4°C. The secondary antibody (Alexa Fluor® 594, anti-rabbit, 1∶1000; Molecular Probes, Invitrogen, Carlsbad, CA) was incubated for 45 min. The primary antibodies used were rabbit anti-hAIPL1 (1∶700; kindly provided by Michael E. Cheetham, University College London, London, UK) and rabbit anti-hCAR [51] (1∶10000; kindly provided by Cheryl M. Craft, University of Southern California, Los Angeles, Ca). Vectashield (Vector Lab Inc., Peterborough, UK) was used to visualize nuclei. Sections were photographed using either a Zeiss Axioplan microscope (Carl Zeiss, Oberkochen, Germany) or a Leica Laser Confocal Microscope System (Leica Microsystems GmbH, Wetzlar, Germany).

### Electroretinography

Electrophysiological recordings in mice were performed as detailed in [52] and bilateral ERG evaluations in pigs were carried out as previously described [38].

### miRNA and gene expression analysis

MiRNA and gene expression analysis in mice administered with the AAV2/5-CMV-*EGFP*-4xmiR124T and AAV2/5-CMV-*EGFP*-4xmiR204T constructs was performed on samples from whole retinas and optic cups, respectively. Total RNA was extracted using the miRNeasy kit (Qiagen, Inc., Hilden, Germany) according to the manufacturer's instructions and quantified using the NanoDrop 1000 (Thermo Fischer Scientific, Waltham, MA). RNA quality was assessed by gel electrophoresis.

Quantitative (q) Reverse Transcriptase (RT-) PCR-based detection of mature miR-124, miR-182 and miR-204 was performed using the TaqMan® microRNA assays (Applied Biosystems, Foster City, CA). All reactions were performed in triplicate. The qRT-PCR results, recorded as threshold cycle numbers (Ct), were converted to absolute copy number (*i.e.* copies of miRNA per ng of RNA) using a standard curve. To generate the standard curve, serial amounts (ranging from 10^2^ to 10^8^ copies) of a synthetic RNA oligonucleotide corresponding to miR-124 (5′-UAAGGCACGCGGUGAAUGCC-3′; Sigma–Aldrich, St. Louis, MO) were mixed with 10 ng of total yeast RNA. The samples were analyzed using the TaqMan® microRNA assay and the correlation between threshold cycle numbers (Ct) and copies of miRNA was established.

For the expression analysis of target genes, cDNA synthesis was performed using the Quantitect Reverse Transcription kit (Qiagen, Inc., Hilden, Germany) starting from 1 µg of DNase-treated RNA. In order to unambiguously distinguish spliced cDNA from genomic DNA contamination, exon-specific primers were designed to amplify across introns of the genes tested. All primers were previously tested by reverse transcription (RT)-PCR and no RT control reactions were performed. Primer sequences are the following: MmRdh10_For: 5′-CTAGAGATTAATCATGGCCAC-3′; MmRdh10_Rev: 5′-CTCGTGAAAACCCACAACTC-3′; MmVamp3_For: 5′-CAGACACAAAATCAAGTAGATG-3′; MmVamp3_Rev: 5′-CAGTGCATCTGCGCGGTC-3′. qRT-PCR experiments were performed using the ABI Prism 7900HT Fast Sequence Detection System with ABI Power SYBR Green reagents (Applied Biosystems, Foster City, CA). Real-time PCR results were analyzed using the comparative Ct method normalized against the housekeeping genes *GAPDH* and *ACTB.* The range of expression levels was determined by calculating the standard deviation of the ΔCT.

## Supporting Information

Figure S1 **AAV vectors harboring miRTs do not detectably perturb miRNA expression and activity in the eye.** (**a**) miRNA expression profile analysis in retinas and optic cups of animals injected subretinally with AAV (n = 4 samples/group). Expression levels were determined by qRT-PCR on RNA extracted from retinas injected with AAV2/5-CMV-*EGFP*-4xmR124T and from optic cups of eyes treated with AAV2/5-CMV-*EGFP*-4xmR204T following delivery of a high AAV vector dose (2.6×10^9^ GC/eye). Subretinal administration of AAV vectors harboring miRTs does not detectably perturb endogenous miRNA expression in the eye. (**b**) Expression levels of *RDH10* and *VAMP3,* two direct targets of miR-124 in retinas injected with high doses of AAV2/5-CMV-*EGFP*-4xmR124T animals (n = 4). The contralateral eyes injected with the AAV2/5-CMV-*EGFP* control were used as reference. *ACTB* and *HPRT* were used as internal controls. Subretinal administration of AAV vectors harboring miRTs does not detectably perturb endogenous miRNA activity in the eye. Error bars represent the mean plus or minus SEM.
(TIF)
