# Supplementary figures and images for: MicroRNA-Restricted Transgene Expression in the Retina
Source: PLoS One. 2011 Jul 26;6(7):e22166. doi: 10.1371/journal.pone.0022166 (PMC3144214; doi:10.1371/journal.pone.0022166)

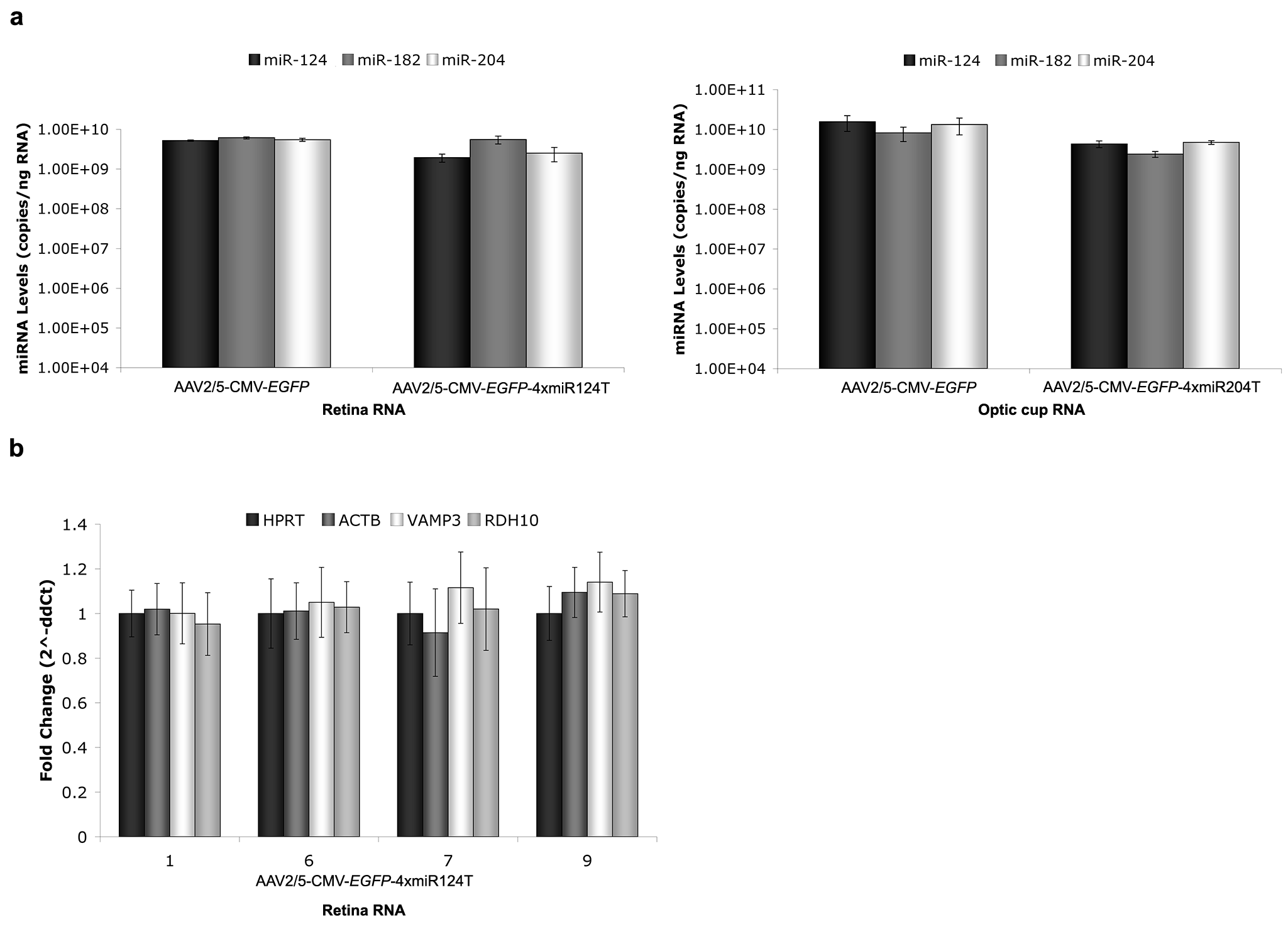

Supplement: Figure S1 — AAV vectors harboring miRTs do not detectably perturb miRNA expression and activity in the eye. (a) miRNA expression profile analysis in retinas and optic cups of animals injected subretinally with AAV (n = 4 samples/group). Expression levels were determined by qRT-PCR on RNA extracted from retinas injected with AAV2/5-CMV-EGFP-4xmR124T and from optic cups of eyes treated with AAV2/5-CMV-EGFP-4xmR204T following delivery of a high AAV vector dose (2.6×10^9^ GC/eye). Subretinal administration of AAV vectors harboring miRTs does not detectably perturb endogenous miRNA expression in the eye. (b) Expression levels of RDH10 and VAMP3, two direct targets of miR-124 in retinas injected with high doses of AAV2/5-CMV-EGFP-4xmR124T animals (n = 4). The contralateral eyes injected with the AAV2/5-CMV-EGFP control were used as reference. ACTB and HPRT were used as internal controls. Subretinal administration of AAV vectors harboring miRTs does not detectably perturb endogenous miRNA activity in the eye. Error bars represent the mean plus or minus SEM. (TIF) [file pone.0022166.s001.tif]
